# Astrocytes differentially respond to inflammatory autoimmune insults and imbalances of neural activity

**DOI:** 10.1186/2051-5960-1-70

**Published:** 2013-10-23

**Authors:** Peter Jukkola, Tomas Guerrero, Victoria Gray, Chen Gu

**Affiliations:** 1 Biomedical Sciences Graduate Program, The Ohio State University, Columbus, OH 43210, USA; 2 College of Medicine, The Ohio State University, Columbus, OH 43210, USA; 3 Department of Molecular Genetics, The Ohio State University, Columbus, OH 43210, USA; 4 Department of Neuroscience, The Ohio State University, 182 Rightmire Hall, 1060 Carmack Road, Columbus, OH 43210, USA

**Keywords:** Experimental autoimmune encephalomyelitis, Astrocytic endfeet, Water channel, Neurovascular coupling, Ankyrin-G, Voltage-gated potassium (Kv) channel

## Abstract

**Background:**

Neuronal activity intimately communicates with blood flow through the blood–brain barrier (BBB) in the central nervous system (CNS). Astrocyte endfeet cover more than 90% of brain capillaries and interact with synapses and nodes of Ranvier. The roles of astrocytes in neurovascular coupling in the CNS remain poorly understood.

**Results:**

Here we show that astrocytes that are intrinsically different are activated by inflammatory autoimmune insults and alterations of neuronal activity. In the progression of experimental autoimmune encephalomyelitis (EAE), both fibrous and protoplasmic astrocytes were broadly and reversibly activated in the brain and spinal cord, indicated by marked upregulation of glial fibrillary acidic protein (GFAP) and other astrocytic proteins. In early and remitting EAE, upregulated GFAP and astrocytic endfoot water channel aquaporin 4 (AQP4) enclosed white matter lesions in spinal cord, whereas they markedly increased and formed bundles in exacerbated lesions in late EAE. In cerebellar cortex, upregulation of astrocytic proteins correlated with EAE severity. On the other hand, protoplasmic astrocytes were also markedly activated in the brains of ankyrin-G (AnkG) and Kv3.1 KO mice, where neuronal activities are altered. Massive astrocytes replaced degenerated Purkinje neurons in AnkG KO mice. In Kv3.1 KO mice, GFAP staining significantly increased in cerebellar cortex, where Kv3.1 is normally highly expressed, but displayed in a patchy pattern in parts of the hippocampus.

**Conclusions:**

Thus, astrocytes can detect changes in both blood and neurons, which supports their central role in neurovascular coupling. These studies contribute to the development of new strategies of neuroprotection and repair for various diseases, through activity-dependent regulation of neurovascular coupling.

## Background

Normal functions of the CNS rely critically on the proper structure and function of the vascular system. Blood vessels provide neurons with oxygen and nutrients and protect them from toxins and pathogens. Neurons, in turn, control blood vessel dilation and contraction. The neurovascular unit consists of neurons, glia (astrocytes, microglia and oligodendrocytes), and vascular cells (endothelia, pericytes and smooth muscle cells). Vascular cells form the BBB and maintain the chemical and cellular composition of the neuronal microenvironment, which is required for proper functioning of neuronal synapses and circuits. Emerging evidence shows some neurodegenerative diseases, such as Alzheimer’s disease, amyotrophic lateral sclerosis, and multiple sclerosis (MS), are initiated and perpetuated by vascular abnormalities [1-3]. Understanding neurovascular coupling will advance the diagnosis, therapy, and prevention of these diseases.

Astrocytes play a key role in neurovascular coupling. Astrocytes outnumber neurons in human brain and spinal cord [4] and fulfill important roles in regulating synapse formation and function [5], and axon myelination [6,7], as well as neurovascular coupling [8]. They are a diverse group of cells in the CNS and can be divided into two major groups. Protoplasmic astrocytes are found in gray matter and their endfoot processes ensheath synapses. Fibrous astrocytes reside in white matter and their endfeet contact myelin membranes and nodes of Ranvier [9]. Endfeet of both types of astrocytes cover more than 90% of brain capillaries [9,10].

MS is an inflammatory demyelinating disease with unknown origin. In brain biopsies of MS patients, lesions are characterized by extensive loss of myelin, axonal damage and inflammation [11-14]. Activation of immune cells and disruption of the BBB are early events in MS pathology and have been extensively studied, but the role of astrocytes in MS progression is relatively unexplored [12]. GFAP was isolated from old MS plaques in 1971 [15]. However, astrocytes only began to gain attention in MS recently due to two channel proteins that are expressed at astrocytic endfeet. The water channel AQP4 regulates water balance, and its auto-antibody can lead to neuromyelitis optica (NMO) [16-18], arguably considered as a variant of MS [19]. In the CNS, AQP4 is predominantly localized to astrocytic endfeet contacting blood capillaries. In NMO, astrocytic AQP4 is the primary target of an immune response that results in profound damage to astrocytes, breakdown of the BBB, secondary loss of myelin, and apoptosis of oligodendrocytes [12,17,20]. An inwardly rectifying K^+^ channel, Kir4.1, is also present in astrocytic endfeet, and is involved in K^+^ buffering [21-23]. Kir4.1 is likely a target of the autoimmune response in a subgroup of MS patients [24]. Our previous studies show that astrocytes in spinal cord white matter are activated during EAE, an animal model for MS [25]. Kv1.4 is upregulated in astrocytes during EAE [25]. How astrocytes are altered in different brain regions is not known. In the past, the astrocyte has been viewed as a cell that promotes inflammation and forms glial scars that hinder remyelination and axon growth. We now know that astrocytes can enhance CNS myelination by promoting the migration, proliferation, and differentiation of oligodendrocyte progenitor cells. Promotion of CNS myelination by astrocytes has been demonstrated in many culture models [6,7,26,27].

Astrocytes express neurotransmitter receptors that allow them to respond to neuronal activity [28]. Astrocytes are activated in epilepsy and ataxia, diseases often caused by imbalances of excitatory and inhibitory neural networks. Glutamate receptors and transporters expressed on astrocytes are altered in epilepsy [29], while a key enzyme in the glutamate cycle, glutamine synthetase, is down-regulated [30,31]. AQP4 polarization to astrocytic endfeet was disrupted in the latent phase of the kainate model of epilepsy, before the onset of spontaneous seizure activity [32]. Astrocyte dysfunction and alterations are involved in other neurodegenerative diseases, including the spinocerebellar ataxia by Ataxin-7 mutant [33]. Therefore, astrocytes play a key role in mediating neurovascular coupling. A better understanding of alterations to neurovascular units in different regions and in response to different stimuli will contribute to our understanding of (1) why certain brain regions are vulnerable to specific insults in certain neurological diseases, and (2) how CNS lesions initiate, extend, and repair in terms of neurons and glia.

In this study, we systematically examined alterations of astrocytic proteins on animal models for different diseases, including MS, neurodegenerative ataxia and epilepsy. Astrocytes are broadly activated in the CNS including both spinal cord and brain by autoimmune inflammation in EAE. On the other hand, altered neuronal activity, particularly synaptic activity, can also activate astrocytes. Thus, astrocytes are a good indicator for alterations in neurovascular coupling and a mediator of the vicious cycles in a number of neurodegenerative diseases.

## Methods

### Reagents and antibodies

The nuclear dye (Hoechst 33342) and the lipophilic dyes (Fluoromyelin-green and Fluoromyelin-red) were purchased from Invitrogen (Carlsbad, CA, USA). The following antibodies were used in our study: rabbit polyclonal anti-Kv1.4, anti-AQP4 (Alomone Labs, Jerusalem, Israel), and anti-PKCγ (Santa Cruz Biotechnology, Dallas, TX); goat polyclonal anti-GFAP (AbCAM, Cambridge, MA, USA); chicken polyclonal anti-Vimentin (Millipore, Temecula, CA); and Dylight 488-, Dylight 649-, Cy3-, and Cy5-conjugated secondary antibodies (Jackson Immuno Research Laboratories, West Grove, PA, USA). Myelin oligodendrocyte glycoprotein (MOG) peptide 35–55 (MEVGWYRSPFSRVVHLYRNGK) was purchased from Pro-Spec (Rehovot, Israel), and proteolipoprotein (PLP) peptide 139–151 (HCLGKWLGHPDKF) from Anaspec (Fremont, CA, USA). Ground inactivated mycobacteria tuberculosis H37Ra and Incomplete Freund’s Adjuvant were from Difco Laboratories (Detroit, MI, USA).

### Induction of chronic EAE

Chronic EAE was induced in 12-week-old female C57BL/6 and Thy1:YFP transgenic mice (Jackson Laboratories, Bar Harbor, ME, USA) according to previously published methods [25]. Briefly, myelin oligodendrocyte glycoprotein (MOG) peptide 35–55 (1 mg/mL final concentration) was emulsified in sterile-filtered PBS and Complete Freund’s Adjuvant (CFA) containing 2 mg/mL (final concentration) ground inactivated mycobacteria tuberculosis H37Ra. Mice were immunized with 100 μL of MOG/CFA or CFA only (control) by subcutaneous injection at four sites in the belly and in each hind footpad. Pertussis toxin was administered by tail-vein injection at 0 and 2 days post immunization (DPI). Disease progression was monitored by daily clinical scoring on a scale of 0–6 [0 = no symptoms, 1 = loss of tail tone, 2 = hindlimb paresis, 3 = moderate paralysis, 4 = paraplegia (complete hindlimb paralysis), 5 = quadriplegia, 6 = death or moribund state]. Grade 6 animals were removed from the study.

### Induction of remitting-relapsing EAE

As previously published [25], procedures for the induction and monitoring of rrEAE in 12-week-old female SJL/J mice (Jackson Laboratories) were identical to chEAE procedures except for the use of a different myelin peptide, PLP 139–151.

### Cardiac perfusion, tissue fixation and sectioning

Animal tissues were collected and processed according to previously published methods, with minor modification [25]. Briefly, mice were deeply anesthetized with avertin and perfused through the heart with 20–30 mL ice-cold PBS followed by 20 mL 4% formaldehyde in PBS (FA/PBS). The brain and spinal cord of EAE mice were carefully removed and post-fixed overnight in 4% FA/PBS. Wildtype, AnkG−/−, and Kv3.1−/− mice were post-fixed in 4% FA/PBS for just 1 h, which allowed improved staining resolution of fine astrocyte processes. Tissues were cryoprotected in 30% sucrose for at least 24 hr, cut into 3-mm blocks using an acrylic brain matrix (Braintree Scientific, Braintree, MA, USA), embedded in optimal cutting temperature (OCT) media (Sakura Finetek USA, Inc., Torrance, CA, USA), and stored at −80°C until sectioning. The tissue blocks were cut with a Microm HM550 cryostat (Thermo Scientific, Waltham, MA, USA) and the 40-μm sections were collected on Superfrost Plus microscope slides (FisherScientific, Pittsburgh, PA, USA) for storage at −20°C.

### Immunofluorescent staining

Tissues were stained according to previously published methods [25], with minor modification. Briefly, sections were permeabilized in PBS/0.4% Triton X-100 for 1 hr at room temperature (RT), blocked with 2.5% normal donkey serum for 1 hr at RT, and incubated in primary antibodies in blocking solution for 3 hr at RT and overnight at 4°C. The next day, the sections were rinsed 10 × 5 min, incubated for 3 hr with secondary antibodies in blocking solution, counterstained in Hoechst 33342 and/or Fluoromyelin for 10 min, and again rinsed 10 × 5 min (all at RT). Slides were coverslipped using tris-buffered Fluoro-Gel mounting media (Electron Microscopy Sciences, Hatfield, PA, USA). Staining for each primary antibody was performed at least twice on tissue from 3–6 mice per experimental cohort, except for the late RREAE cohort in which only 2 mice were available.

### Fluorescence light microscope and image capture

Fluorescence microscopy and image analysis procedures were adapted from previously published methods [25,34,35]. Images were captured with a Spot CCD camera RT slider (Diagnostic Instruments, Sterling Heights, MI, USA) in a Zeiss Axiophot upright microscope using10×/0.30 and 20×/0.50 Plan Apo objectives and saved as 16-bit TIFF files. Exposure times were controlled so that the pixel intensities in the tissues of interest were below saturation, and the same exposure time was used for each group within an experiment. Image brightness and contrast were adjusted using Adobe Photoshop 7.0 (Adobe Systems Incorporated, San Jose, CA, USA).

Images to be quantified were chosen from 2 representative experiments for each antibody, and compared between experimental cohorts (3 mice quantified per cohort). The number of images quantified is noted on the bar graphs in each figure. Images were analyzed with MetaMorph (Molecular Devices, Downingtown, PA, USA) and Sigmaplot 11.0 (Systat Software, Inc., Chicago, IL, USA) for fluorescence intensity quantification and statistical testing. Staining intensities for GFAP, Vimentin, Kv1.4, and AQP4 were measured by using the MetaMorph region measurement tool to sample and automatically calculate the average pixel fluorescence intensities of small circles (~ 50-pixel area) drawn on astrocyte soma and processes (10–20 sampled per image), and these intensities were averaged for each image. The background intensity of the slide was then measured and subtracted from the astrocyte staining intensity for each image. To account for variations in staining intensity from images obtained in different staining experiments, the data were normalized to controls stained side-by-side. The image fluorescence intensity values were averaged to obtain the mean fluorescence intensity for each experimental group, expressed as mean ± SEM. These fluorescence intensity measurements reflect the level of protein present within the cells stained by a particular antibody (higher intensity correlates with more protein), and do not give any indication of lesion size or cell number. To quantify cell numbers in hippocampus or cortex, the area of the region of interest in each image was first measured using the MetaMorph region measurement tool. Then, the GFAP + cells present in these regions were counted manually and expressed as the number of cells per mm^2^, mean ± SEM. Statistical significance was determined between two groups using an unpaired Student’s *t* test, and among three or more groups using One-Way ANOVA followed by Fisher’s test. Statistically significant differences from the Control group (for EAE experiments) or the Wildtype group (for KO mice) are shown by an asterisk (*) in figures. All fluorescence intensity measurements were taken from the originally captured images.

### Confocal microscopy and 3D reconstruction

High-magnification confocal images were captured with a Leica TCS SL confocal imaging system (Leica Microsystems, Mannheim, Germany), using a 100× HCX Plan Apo CS oil immersion objective (numerical aperture = 1.40). Multiple channels were acquired simultaneously, or sequentially only if needed to eliminate channel bleed-through. Channel bleed-through was largely eliminated through optimization of the laser line intensity by acousto-optical tunable excitation filters, and by spectral detectors allowing precisely-defined bandwidth adjustment. The signal was averaged over eight scans in linescan mode. Images were saved as 8-bit TIFF files and adjusted for brightness and contrast using Adobe Photoshop 7.0.

For 3D reconstruction, z-stacks of images were collected with a 0.5-μm slice interval. Collapsed images were created using a maximum intensity projection of the z-stack. For each 3D image, the z-stack was visualized in three-dimensional cross section, and the cross-bars were centered on pertinent features of the image. The three one dimensional images were then exported together as an 8-bit TIFF file. Supplemental movies were created using the Maximum Projection with Animation tool within the Leica Confocal Software (v2.61). A maximum intensity projection of the z-stack was rotated 90 degrees around the Y-axis in a series of 18 or 36 steps and exported as an AVI file, and converted to an MPEG-1 file (MPG).

### AnkG and Kv3.1 KO mice

AnkG KO mice were previously published [36]. The Kv3.1 knockout (KO) mouse line was kindly provided by Dr. R. Joho at UT Southwestern Medical Center and has been maintained using a PCR-based genotyping procedure as previously described [37-39]. The Kv3.1 KO mice were backcrossed with C57BL/6 for ten generations. Although very rare, seizure convulsions (last only 10–15 sec; mouse returns to normal within a few minutes) can be observed occasionally from some Kv3.1 homozygote (−/−) but not heterozygote (+/−) mice. The following primers were used: forward primer 31 F775 (for both WT and knockout, 5′- GCG CTT CAA CCC CAT CGT GAA CAA GA -3′), reverse primer 31R991 (for WT, 5′- GGC CAC AAA GTC AAT GAT ATT GAG GG -3′), and reverse primer PNR278 (for knockout, 5′- CTA CTT CCA TTT GTC ACG TCC TGC AC -3′). Three Kv3.1 KO (−/−) and three control C57BL/6 mice of either sex at the age of 2–4 months were used in this study.

All animal experiments have been conducted in accordance with the NIH Animal Use Guidelines and were approved by the Ohio State University Institutional Animal Care and Use Committee (IACUC).

## Results

### AQP4, Kv1.4, Vimentin and GFAP are upregulated in SCWM at different stages of EAE

**Figure 1:**
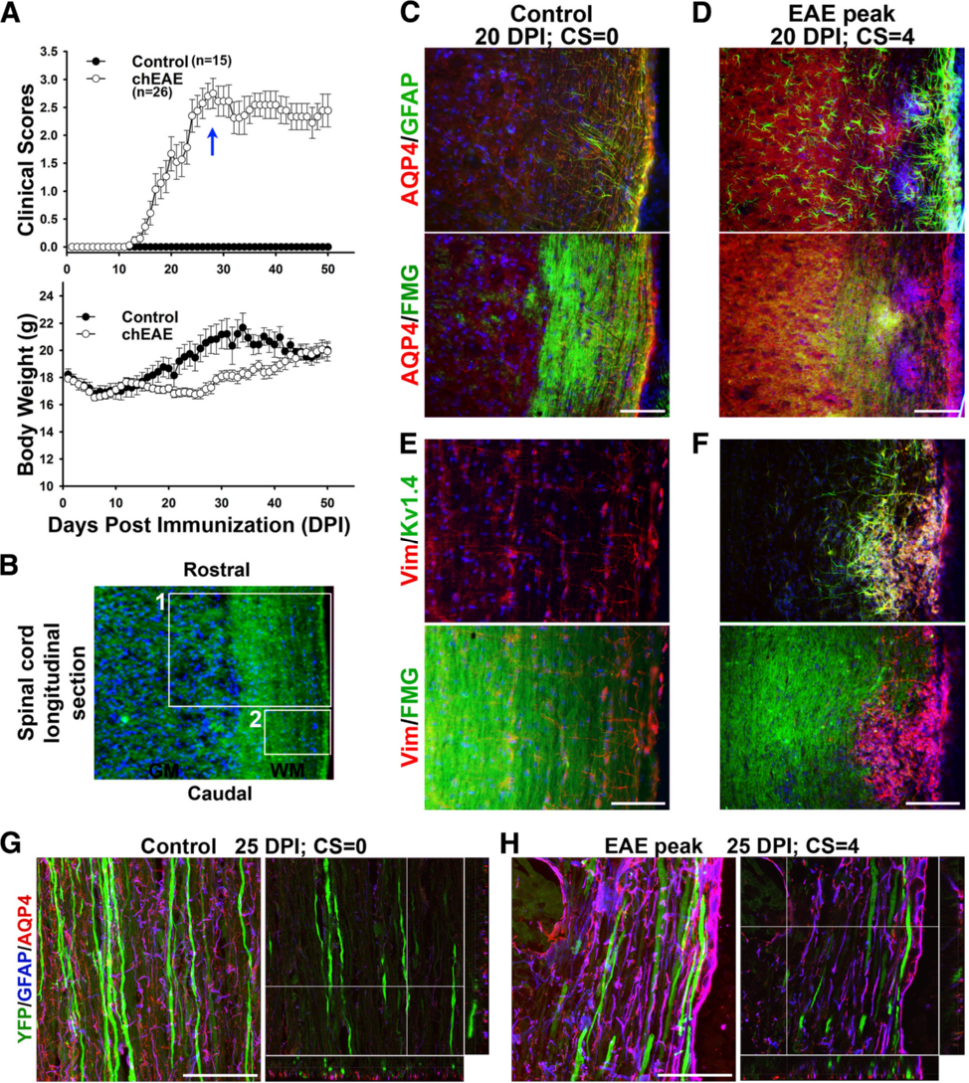
**Activation of astrocytes in spinal cord white matter. A**, Clinical scores (top) and body weight (bottom) of mice with chEAE. **B**, White matter (WM) and gray matter (GM) in spinal cord longitudinal section was stained with FMG (green) and nuclear dye (blue). Box 1 shows both GM and WM and box 2 shows only WM. Spinal cord sections, control **(C)** and EAE peak **(D)**, were co-stained for GFAP (green, top), AQP4 (red), Hoechst (blue), and FMG (green, bottom). Co-staining of Kv1.4 (green, top), Vim (red), Hoechst (blue) and FMG (green, bottom) were also performed on control **(E)** and EAE **(F)** spinal cord sections. High magnification confocal image stacks were obtained from control **(G)** and EAE **(H)** Thy1-YFP transgenic mice. Images contain YFP (green), GFAP (blue) and AQP4 (red). The collapsed 2D image is on the left, and 3 cross sections are on the right. In **(G)**, the crossbars are centered on a putative node of Ranvier. In **(H)**, the crossbars are centered on the AQP4+/GFAP + lesion edge. Scale bars, 500 μm in **C-F**, 50 μm in **G**,**H**.

To understand how inflammatory autoimmune insults affect astrocytes in the CNS, we performed the MOG_35-55_-induced chronic EAE (chEAE) (Figure 1A), as previously described [25]. First, we examined spinal cord white matter (SCWM) and gray matter (SCGM). In SCWM of control mice, AQP4 and Vimentin (Vim) were colocalized in GFAP-positive astrocytes (Figure 1C,E). At the peak stage of chEAE, GFAP was significantly upregulated around demyelinated lesion sites, where AQP4 was also upregulated (Figure 1D). The Kv1.4 level was upregulated together with Vim around the lesion sites (Figure 1E,F). Both GFAP and Vim are upregulated in reactive astrocytes. Although both are intermediate filaments, they differ in temporal and spatial expression patterns in astrocytes. Vim is expressed in radial glia and immature astrocytes, as well as other cell types, whereas GFAP replaces Vim in many mature astrocytes [40-42]. In adult CNS, Vim expression may indicate astrocyte proliferation. In the present study, we use it as a marker for distinct subpopulations of astrocytes. In our recent study, we found that Kv1.4 was upregulated in astrocytes of SCWM near the EAE lesion sites [25], but not in astrocytes in other regions of the CNS. Interestingly, both Kv1.4 and Vim expression were upregulated in SCWM (Figure 1E,F). Therefore, we examined Vim expression in other regions of the CNS during EAE in following studies, which may suggest potential astrocytic proliferation.

Using high magnification confocal imaging, we examined alterations of the neurovascular unit (axons, astrocytes, and capillaries) of Thy1-YFP transgenic mice. Axons were revealed by YFP fluorescence, astrocytes were stained for GFAP, and capillaries were revealed with AQP4 staining. In control, fibrous astrocytes in SCWM extended processes parallel with axons, while AQP4 in astrocyte endfeet colocalized with putative nodes of Ranvier along YFP-positive axons (Figure 1G and Additional file 1: Movie S1). At the peak stage of chEAE, GFAP and AQP4 staining disappeared in the center of the lesion, but was often upregulated surrounding lesion sites (Figure 1H and Additional file 2: Movie S2). YFP-positive axons were often fragmented near the lesion, indicating axonal degeneration (Figure 1H and Additional file 2: Movie S2). EAE lesions were only observed in WM but not in GM.

### Differential alterations of myelin and astrocytes in the late and remitting EAE in SCWM

**Figure 2:**
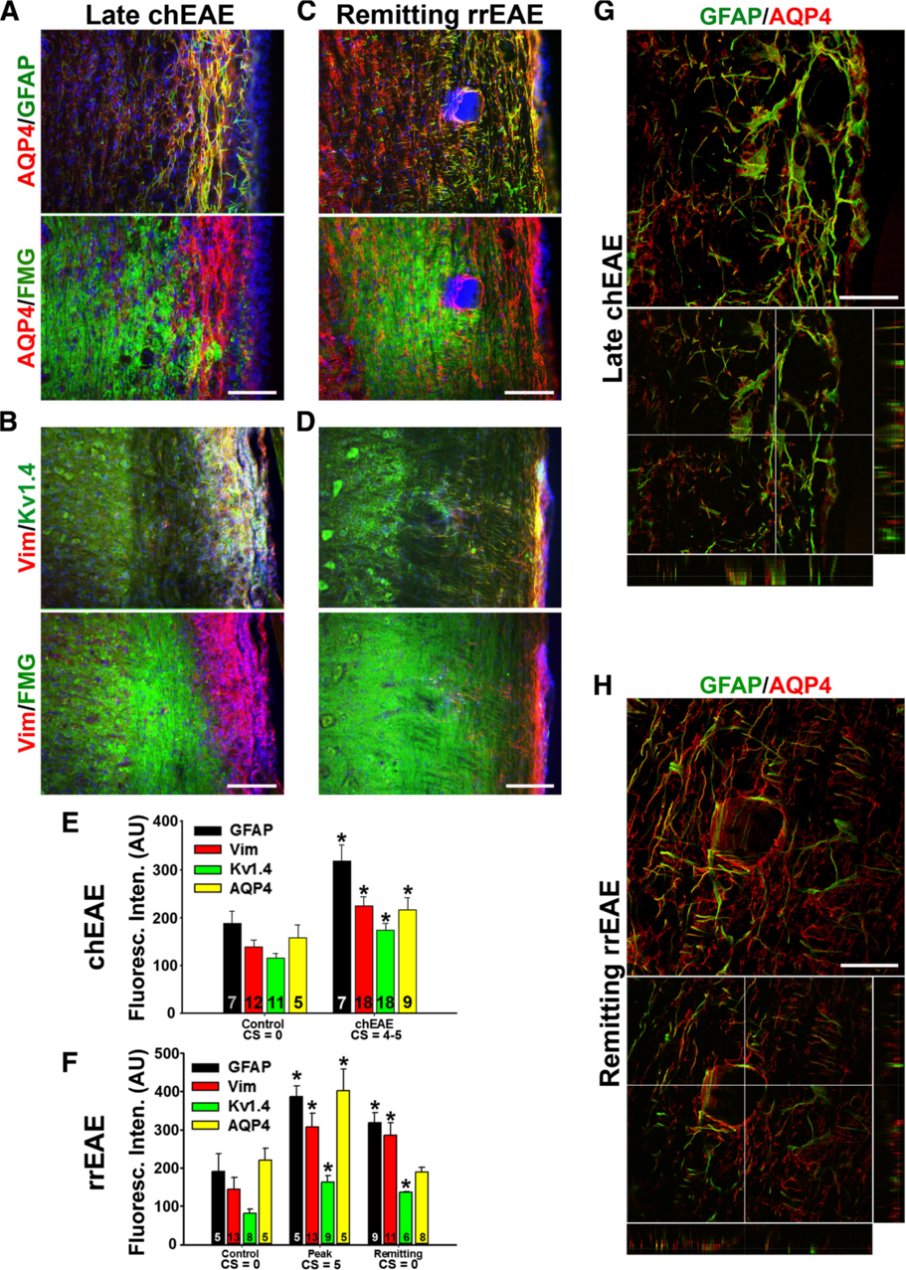
**Astrocytic proteins are altered in lesion sites of at the late stage of chEAE and at the remitting stage of rrEAE. A** and **B**, staining patterns of astrocytic proteins at the late stage of chEAE. **C** and **D**, staining pattern of astrocytic proteins at the remitting stage of rrEAE. **E**, Summary of the alteration of astrocytic proteins in chEAE. **F**, Summary of the alteration of astrocytic proteins in rrEAE. The “n” number of quantified images is provided for each bar. One-way ANOVA followed by Fisher’s test. Asterisk (*) shows significant difference from Control staining intensity for each antibody, *p* < 0.05. **G**, High magnification view of a lesion site at the late stage of chEAE. **H**, Confocal images of a lesion site at the remitting stage of rrEAE. Collapsed 2D image is on the top, and 3 cross sections are on the bottom. In **(G)**, the crossbars are centered on an astrocyte with colocalizing AQP4 and GFAP. In **(H)**, the crossbars emphasize the absence of AQP4 and GFAP in the lesion core. Scale bars, 500 μm in **A-D**, 50 μm in **G**,**H**.

We further compared altered protein expression in astrocytes at the late stage of chEAE and at the remitting stage of remitting-relapsing EAE (rrEAE). In late chEAE, demyelinated lesion areas were very extensive. Both AQP4 and GFAP colocalized very well within and around the lesion sites (Figure 2A,E). Kv1.4 and Vim levels also markedly increased in these dense astrocytic fibers (Figure 2B,E). In contrast, lesions in remitting rrEAE were fewer and much smaller, and enclosed by GFAP- and AQP4-positive processes (Figure 2C). The increase of Kv1.4, Vim and GFAP but not AQP4 levels were significant at the remitting stage (Figure 2C,D,F). High magnification confocal images revealed GFAP staining in extended processes of astrocytes, colocalizing with AQP4 in lesion sites at the late stage of chEAE (Figure 2G). In contrast, at the remitting stage of rrEAE, a small lesion was fully enclosed by GFAP- and AQP4-positive processes (Figure 2H). This result indicates that activated astrocytes may deter the infiltration, proliferation and migration of immune cells to limit the lesion at the initial and remitting stages of EAE.

### Cerebellar astrocytes are differentially activated at different stages of EAE

**Figure 3:**
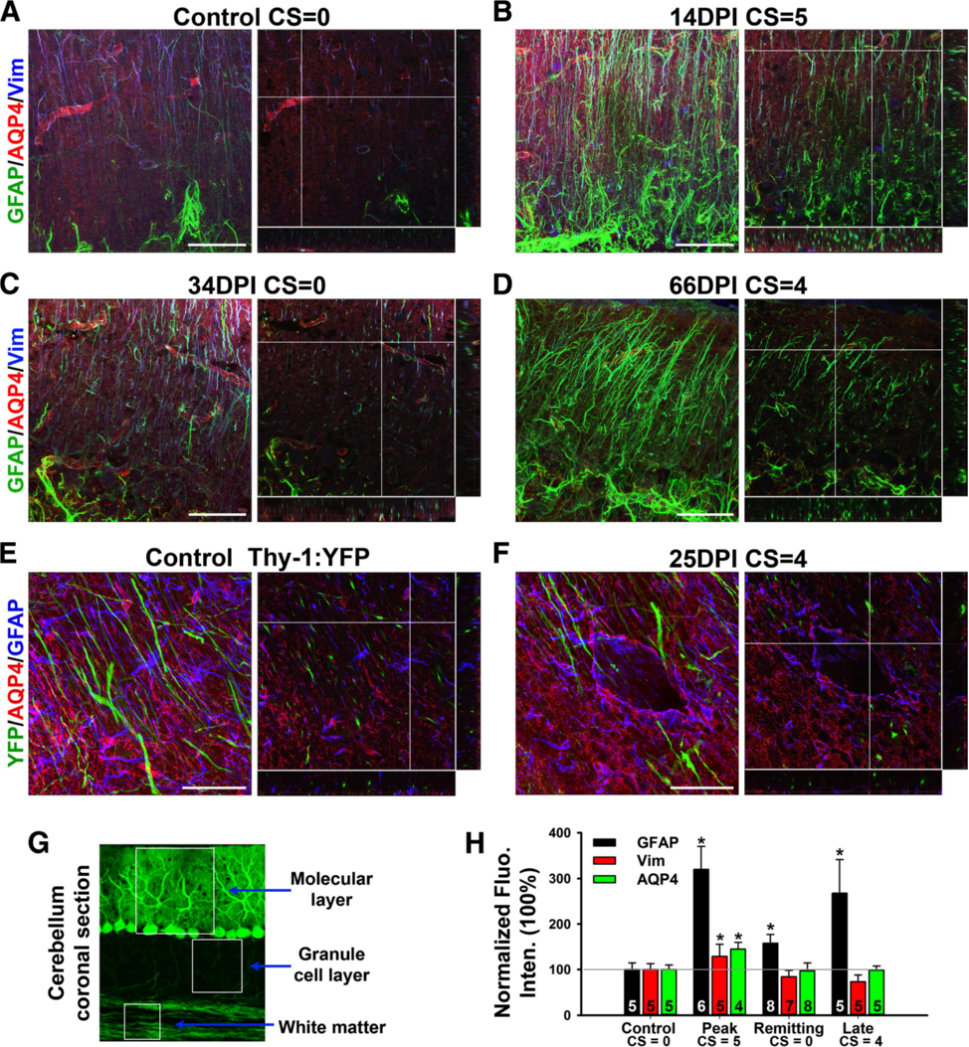
**Differentially altered expression of astrocytic proteins in the cerebellum of EAE mice. A**, The confocal image stack of cerebellar molecular layer that was stained for GFAP (green), AQP4 (red) and Vim (blue) from a control mouse. Collapsed 2D image is on the left and 3 cross sections of 3D are on the right. **B**, The images at the peak stage of an rrEAE mouse. **C**, The images at the remitting stage of an rrEAE mouse. **D**, The images at the relapsing stage of an rrEAE mouse. **E**, The confocal images of cerebellar WM from a Thy1-YFP transgenic mouse. YFP (green), AQP4 (red) and GFAP (blue). In **(A-E)**, the crossbars are centered on astrocytic endfeet with colocalizing AQP4 and GFAP. **F**, The confocal images at the peak stage of chEAE. The crossbars show the lesion edge with upregulated AQP4 and GFAP. **G**, Structural diagram of cerebellar cortex. **H**, Summary of changes of protein levels during rrEAE in cerebellar molecular layer. One-Way ANOVA followed by Fisher’s test. *, significant difference from Control for each antibody, *p* < 0.05. The “n” number of quantified images is provided for each bar. Scale bars, 50 μm.

EAE lesions were observed in cerebellar white matter, especially in EAE mice with high clinical scores [25]. We wondered whether cerebellar gray matter including various astrocytes is affected during EAE. In the cerebellum of control mice, GFAP was expressed at relatively low levels in WM (highest), granule cell layer (medium), and molecular layer (lowest) (Bergmann glia). In the molecular layer, Bergmann glia expressed both GFAP and Vim at low levels, whereas protoplasmic astrocytes surrounding Purkinje neuron soma expressed higher levels of GFAP but with no expression of Vim (Figure 3A and Additional file 3: Movie S3). AQP4 was expressed rather diffusedly but concentrated around blood capillaries (Figure 3A and Additional file 3: Movie S3). At the peak stage of rrEAE, the GFAP level significantly increased in both Bergmann glia and protoplasmic astrocytes (Figure 3B,H and Additional file 4: Movie S4). At the remitting stage, GFAP levels reduced (Figure 3C,H and Additional file 5: Movie S5). At the relapsing stage, GFAP staining increased again (Figure 3D,H and Additional file 6: Movie S6). The Vim level in the Bergmann glia did not change much, and it was not expressed in the protoplasmic astrocytes (Figure 3). The AQP4 level only moderately increased at the peak stage of rrEAE (Figure 3). Therefore, astrocytic GFAP levels in cerebellar gray matter are altered during EAE, in addition to the changes in cerebellar WM, where EAE lesions were observed sometimes. The alterations of neurovascular units in cerebellar WM were similar to those of SCWM, except that all lesion sites in cerebellar white matter were enclosed, overall smaller than those in SCWM (Figure 3E,F). AQP4 was more concentrated in the granule cell layer. Its expression increased around lesions in cerebellar WM. In the molecular layer, its expression was around the blood vessels.

Fibrous GFAP + and AQP4+ processes, and axons were observed in SCWM and white matter of cerebellum and corpus callosum. Their organization is quite similar in all of these areas. Although upregulation of GFAP was also observed in corpus callosum, no lesion was observed there at any stage of EAE (data not shown).

### Hippocampal astrocytes are activated at the peak and late stages of EAE

**Figure 4:**
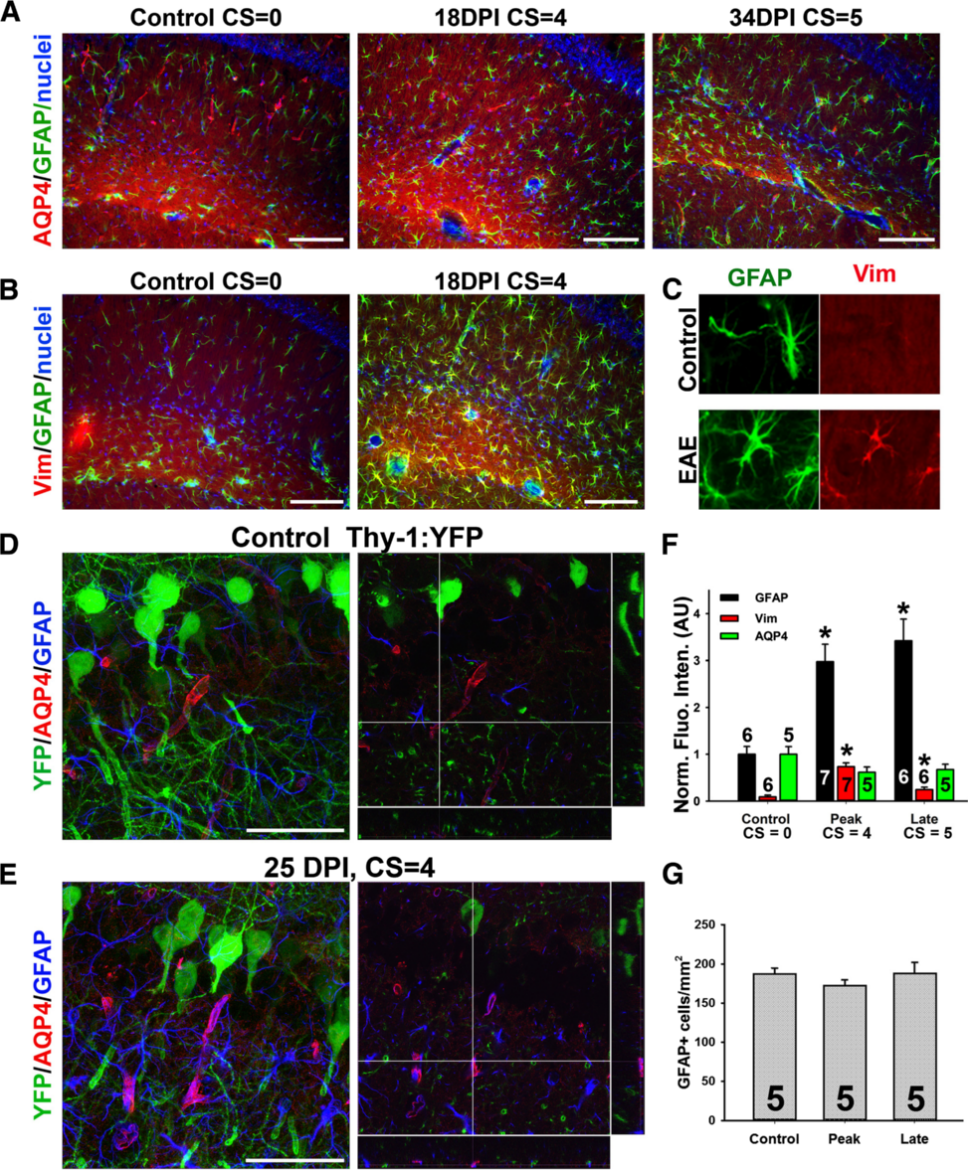
**Activation of astrocytes in the hippocampus of EAE mice. A**, Increased GFAP but not AQP4 staining in the hippocampus of EAE mice. **B**, Increased Vim staining in the hippocampus during EAE progression. **C**, Enlarged images of individual astrocytes in the hippocampus clearly show the increase of Vim staining. High magnification confocal image stacks were obtained from control **(D)** and EAE **(E)** Thy1-YFP transgenic mice. Images contain YFP (green), GFAP (blue) and AQP4 (red). The collapsed 2D image is on the left, and 3 cross sections are on the right. The crossbars reveal astrocytic endfeet with colocalizing AQP4 and GFAP. **F**, Summary of the levels of astrocytic proteins at different stages during EAE progression. One-Way ANOVA followed by Fisher’s test, *, significant difference from Control for each antibody, *p* < 0.01. **G**, GFAP + cell number was not changed during EAE. The “n” number of quantified images is provided for each bar. Scale bars, 200 μm in **A** and **B**, 50 μm in **D** and **E**.

Although no lesion was clearly observed in the hippocampus during the course of EAE, we surprisingly observed the upregulation of GFAP staining in the hippocampus. GFAP-positive protoplasmic astrocytes were observed in the hippocampi of control mice, providing non-overlapping but complete coverage of the entire hippocampus (Figure 4A). During EAE, GFAP levels markedly increased, while AQP4 levels did not change (Figure 4A). In contrast to SCWM astrocytes and Bergmann glia, hippocampal astrocytes normally do not express Vim, but during EAE its expression level significantly increased (Figure 4B,C,F). We further examined the changes of the neurovascular unit within the hippocampus using the Thy1-YFP transgenic mice. In the control, the hippocampal neuron soma, dendrites and axons were revealed by YFP fluorescence. Astrocytes were stained with GFAP antibody, and AQP4 staining revealed ensheathed blood capillaries (Figure 4D and Additional file 7: Movie S7). During EAE, neuronal morphology including axons, dendrites and dendritic spines, remained largely unchanged, whereas GFAP staining markedly increased. The level of AQP4 did not change, but perivascular GFAP astrocyte staining significantly increased (Figure 4E and Additional file 8: Movie S8).

### Cortical astrocytes are activated at the peak and late stages of EAE

**Figure 5:**
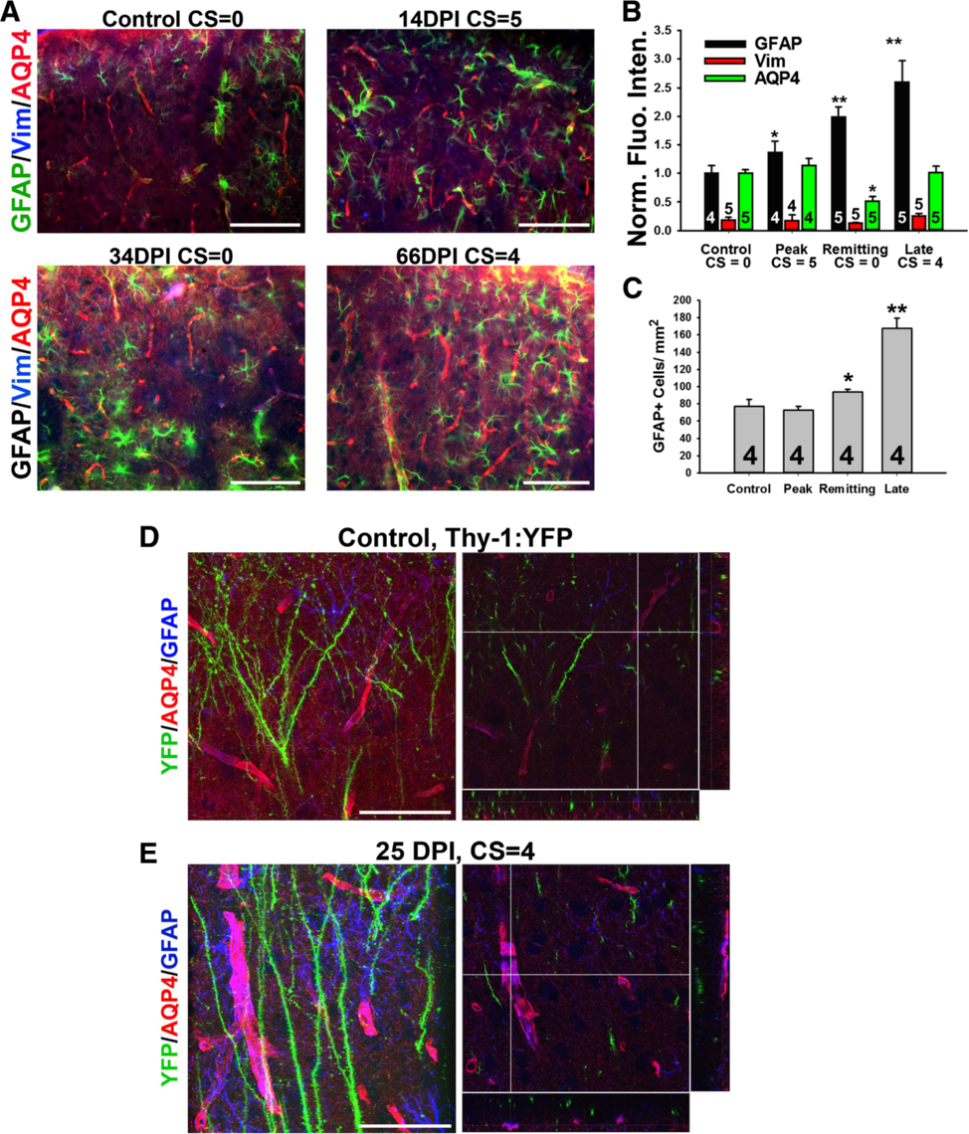
**Alteration of GFAP and AQP4 in the cortex of EAE mice. A**, Alterations of astrocytic proteins in the cortex at different stages of rrEAE. **B**, Summary of the alterations of intensities. **C**, Summary of increased GFAP + cells. **(B, C)** One-Way ANOVA followed by Fisher’s test. *, significant difference from Control for each antibody, *p* < 0.05. **, significant difference from Control for each antibody, *p* < 0.01. The “n” number of quantified images is provided for each bar. High magnification confocal image stacks were obtained from control **(D)** and EAE **(E)** Thy1-YFP transgenic mice. Images contain YFP (green), GFAP (blue) and AQP4 (red). The collapsed 2D image is on the left, and 3 cross sections are on the right. Scale bars, 200 μm in **A**, 50 μm in **D** and **E**.

In the cortex of control mice, GFAP staining was relatively weak, almost invisible in some areas. Like in the hippocampus, AQP4 staining was mainly concentrated around small-diameter blood capillaries. At the peak stage of rrEAE, GFAP markedly increased, whereas AQP4 did not appear to change much. At the remitting stage of rrEAE, GFAP staining was significantly increased. At the relapsing stage of rrEAE, GFAP staining intensity further increased (Figure 5A,B). Perivascular GFAP appeared increased significantly (Figure 5A). There was no Vim staining in the cortex, even in EAE progression, indicating that the properties of cortical astrocytes are different from those of hippocampal astrocytes, even though they are all protoplasmic astrocytes in GM.

Next, we performed high-resolution confocal imaging of the cortex using Thy1-YFP mice. The dendrites and spines were clearly seen in cortical pyramidal neurons (Figure 5D and Additional file 9: Movie S9). Astrocytes and blood vessels intertwined with neurons. Dendritic spines partially colocalized with background AQP4, which possibly represented the synapse-ensheathing endfeet of astrocytes. Astrocytic endfeet are known to colocalize with synapses. Dendritic spines are the postsynaptic structures of excitatory synapses. In the cortex of EAE mice, dendritic branches and spines were not changed, but GFAP intensity and the number of GFAP + astrocytes increased (Figure 5B,C,E and Additional file 10: Movie S10). In particular, the GFAP + processes surrounding blood capillaries positive for AQP4 appeared to increase significantly and capillaries appeared dilated (Figure 5E and Additional file 10: Movie S10). Thus, cortical astrocytes in EAE are primarily altered at the perivascular endfeet. The dendritic spines and surrounding AQP4 staining puncta do not appear to be altered (Figure 5D,E).

### Astrocyte alterations in AnkG and Kv3.1 KO mice

To understand how neuronal activity regulates astrocyte and BBB function, we focused our studies on AnkG and Kv3.1 KO mice. Crucial for efficient initiation and saltatory propagation of action potentials along myelinated axons of vertebrates, Nav channels are clustered at the axon initial segment and nodes of Ranvier [43,44]. The clustering of Nav channels, as well as some K^+^ channels and cell adhesion molecules at axon initial segments and nodes, is mediated by AnkG [36,45-48]. AnkG links these key membrane proteins to the actin cytoskeleton via spectrins and functions as a gate to maintain the axon-dendrite polarity [49,50]. Since most nodes in cerebellar WM have no adaptor protein AnkG [36], the clustering of Nav channels at these nodes is eliminated and thereby nodal excitability is altered, which may conversely impact the astrocytic endfeet around the nodes. It has never been shown before how astrocytes are altered in AnkG KO mice.

The Kv3 (Shaw) channel subfamily contains 4 members (Kv3.1 to Kv3.4). Kv3.1 and Kv3.2 carry sustained currents, while Kv3.3 and Kv3.4 carry transient currents. Kv3 channels play a critical role in rapid spiking of action potentials in some neurons [51-53]. Axonal targeting and unique channel biophysical properties of Kv3 channels—high activation threshold, fast activation/deactivation kinetics—allow neurons to fire action potentials at high frequency [51,54-59]. KO mice were generated to study the functions of different Kv3 channels. Kv3.1 KO mice are viable and fertile [37]. Their spontaneous locomotor and exploratory activities remain unchanged, although their coordinated motor skill and muscle contraction are worsened. Kv3.1 is expressed in cerebellar granule cells and deep nuclei neurons, which are critical for timing or gait patterning in motor functions [38,60,61]. Kv3.1b has also been shown to localize in some nodes of Ranvier in spinal cord white matter [62]. We were interested to determine how alteration of neuronal activity in the absence of AnkG or Kv3.1 would affect astrocytes and BBB function.

**Figure 6:**
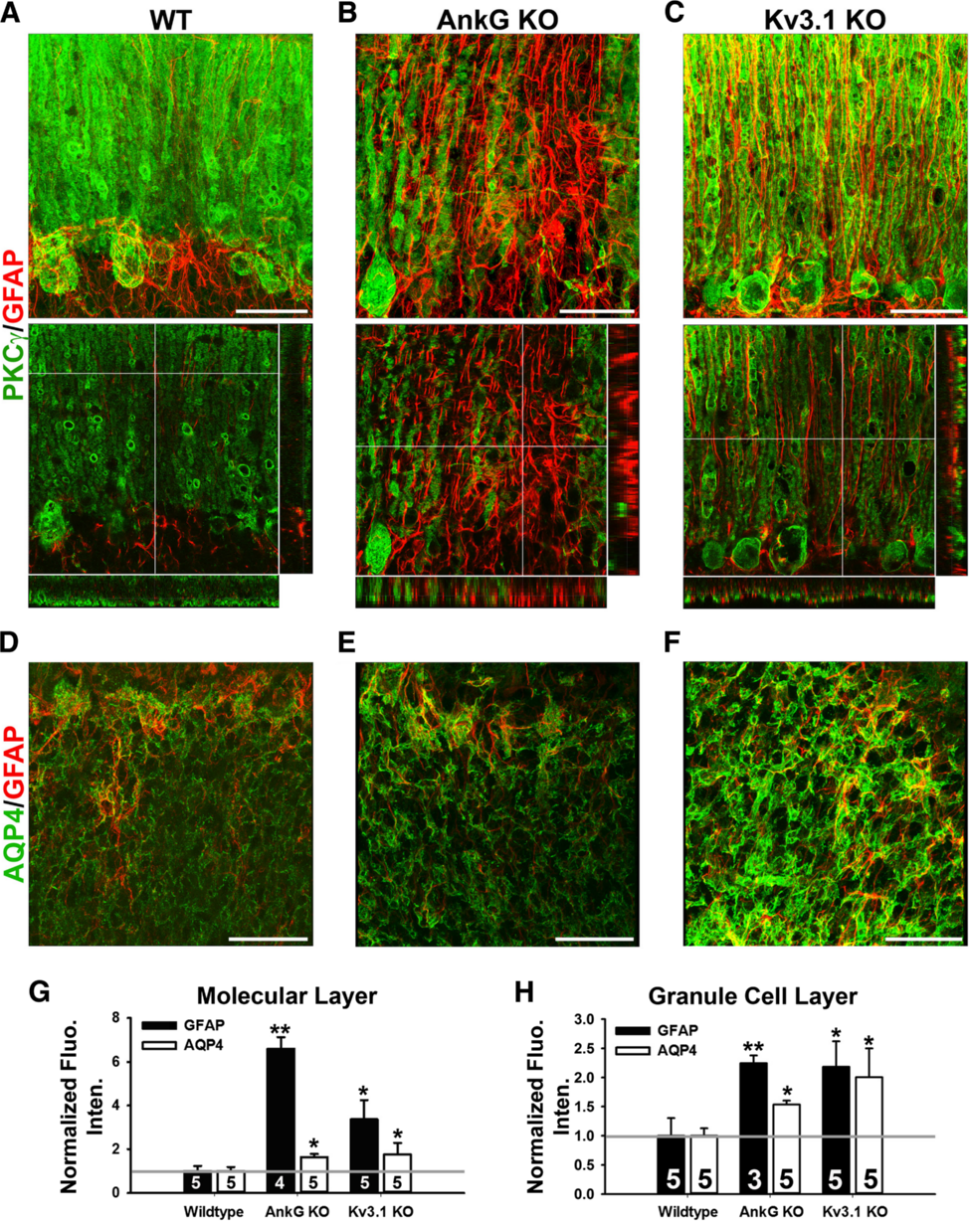
**Upregulation of GFAP and AQP4 in the cerebellum in AnkG and Kv3.1 KO mice. A**, High magnification image stacks of cerebellar molecular layer stained with anti-PKCγ (green) and anti-GFAP (red) antibodies. The collapsed 2D image is on the left, and 3 cross sections are on the right. **B**, Confocal image stacks from the AnkG KO mice. **C**, Confocal image stacks from the Kv3.1 KO mice. In **(A,C)**, the crossbars reveal radially oriented GFAP + Bergmann glial processes in **(A)** WT and **(C)** Kv3.1 KO mice. In **(B)**, the crossbars are centered on highly upregulated GFAP + astrocyte processes in the absence of Purkinje neurons in an AnkG KO mouse. **D**, A single confocal image of the granule cell layer in a WT mouse. **E**, An image from the AnkG KO mice. **F**, An image from the Kv3.1 KO mice. **G**, Normalized fluorescence intensity in the molecular layer. **H**, Normalized fluorescence intensity in the granule cell layer. **(G, H)** One-Way ANOVA followed by Fisher’s test. *, significant difference from Wildtype for each antibody, *p* < 0.05. **, significant difference from Wildtype for each antibody, *p* < 0.01. The “n” number of quantified images is provided for each bar. Scale bars, 50 μm.

Since the AnkG KO is missing the AnkG isoform primarily expressed in the cerebellum and Kv3.1 is highly expressed in the granule cells in the cerebellum, we focused our studies on the cerebellum. In AnkG KO mice, GFAP was increased in the cerebellar molecular layers, from high to low in the following order, molecular layer, Purkinje cell layer, and granule cell layer. PKCγ in WT mice labeled the Purkinje neurons including soma and dendrites (Figure 6A and Additional file 11: Movie S11). In the AnkG KO mice, dendritic branches of some Purkinje neurons reduced, and some Purkinje neurons were degenerated, where GFAP staining signals markedly increased (Figure 6B and Additional file 12: Movie S12). These signals likely result from both increased GFAP expression and increased number of astrocytes. The increased GFAP-positive astrocyte processes were not as oriented as the regular Bergmann glia cells (Figure 6B and Additional file 12: Movie S12). In Kv3.1 KO mice, GFAP signal intensity also significantly increased in the molecular layer of the cerebellum (Figure 6C,G and Additional file 13: Movie S13). In summary, in the molecular layer both GFAP and AQP4 staining increased in both AnkG and Kv3.1 KO mice (Figure 6G). We next examined the granule cell layer. In AnkG KO mice, GFAP and AQP4 staining in granule cell layer were higher (Figure 6E,H). In Kv3.1 KO mice, both GFAP and AQP4 staining was higher (Figure 6F,H).

**Figure 7:**
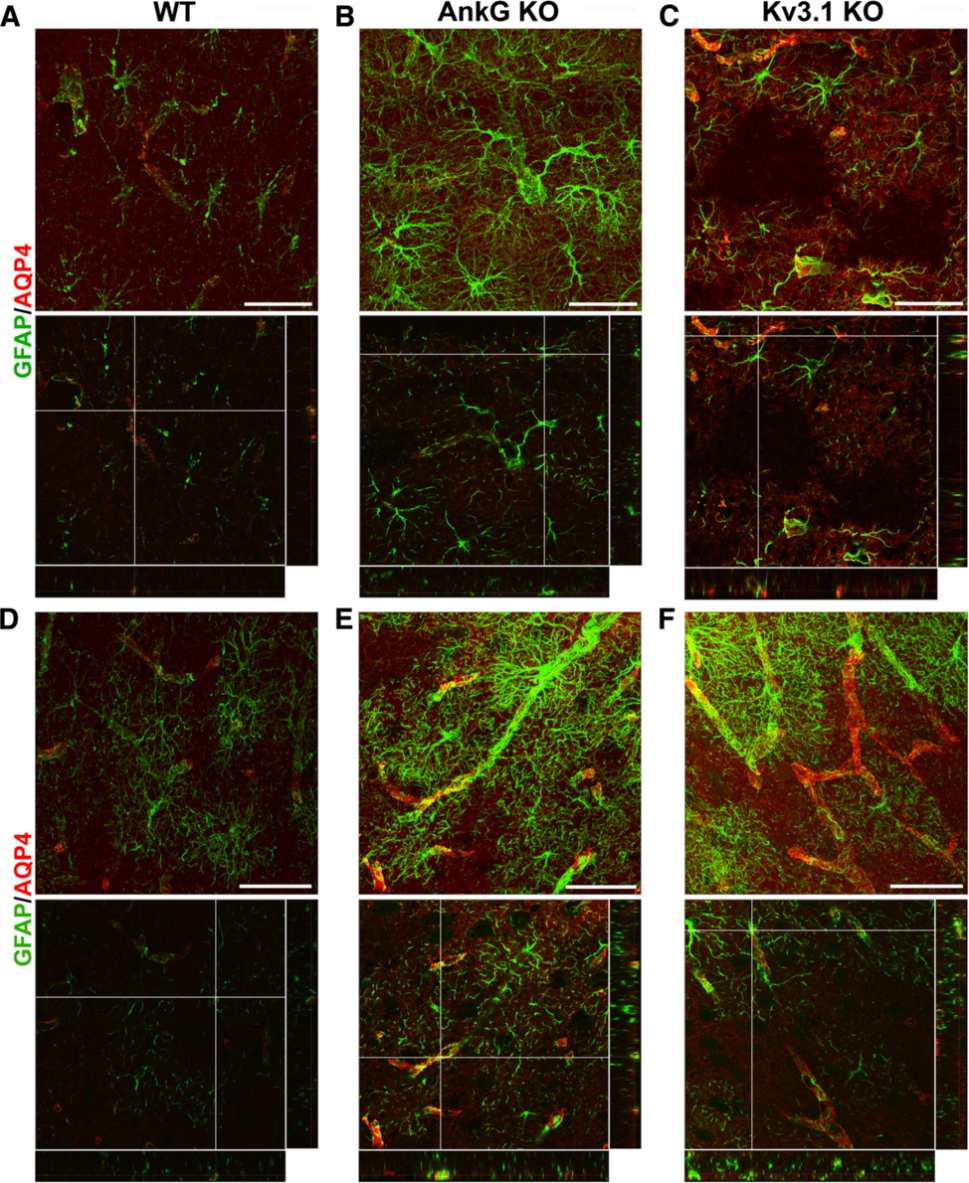
**Altered astrocytes in the hippocampus and cortex in Kv3.1 KO mice.** The confocal image stacks of hippocampus **(A-C)** and cortex **(D-F)** were costained for GFAP (green) and AQP4 (red) from WT **(A,D)**, AnkG KO **(B,E)** and Kv3.1 KO **(C,F)** mice. The collapsed 2D image is on the top and 3 cross sections are at the bottom. The crossbars are centered on astrocyte endfeet with colocalizing AQP4 and GFAP. Scale bars, 100 μm.

Finally, we examined astrocytic proteins in the hippocampus and cerebral cortex. In the hippocampi of AnkG and Kv3.1 KO mice, GFAP staining intensity was increased (Figure 7A-C and Additional file 14: Movie S14, Additional file 15: Movie S15 and Additional file 16: Movie S16). Interestingly, empty pockets of GFAP and AQP4 staining were found in Kv3.1 KO mice (Figure 7C), potentially indicating the death of some astrocytes. In the cortex of AnkG KO and Kv3.1 KO mice, GFAP staining intensity was also increased, but in a non-uniform way (Figure 7D-F and Additional file 17: Movie S17, Additional file 18: Movie S18 and Additional file 19: Movie S19). The density of the brain capillary network appeared increased in both the hippocampus and cortex in Kv3.1 KO mice (Figure 7C,F).

## Discussion

**Figure 8:**
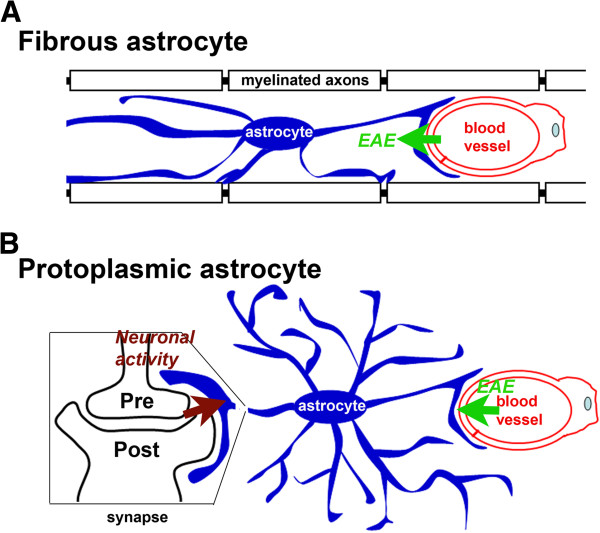
**Diagram of regulation of fibrous and protoplasmic astrocytes by inflammation and neuronal activities. A**, Fibrous astrocytes in the WM with endfeet contacting blood capillaries and nodes of Ranvier. **B**, Protoplasmic astrocytes in the GM with endfeet contacting blood capillaries and synapses.

In this study, we show alterations of neurovascular units in different regions of the brain and spinal cord in response to inflammation and altered neuronal activity. The analysis of neurovascular units has been carried out with high-resolution confocal imaging and 3D reconstruction. This study mainly focuses on astrocytic proteins and provides subcellular structural details regarding disease progression induced by either inflammatory insults or neuronal defects. Our studies indicate striking intrinsic heterogeneity of astrocytes in the CNS, which likely underlies their differential responses to various stimuli (Figure 8).

### Both fibrous and protoplasmic astrocytes are activated during EAE, but only fibrous astrocytes are selectively damaged around lesion sites

The organization of fibrous astrocyte, endfeet AQP4 and axons is quite similar in the WM of spinal cord, cerebellum, and corpus callosum. GFAP and AQP4 were upregulated during EAE. During EAE progression, lesions were observed in WM of spinal cord and cerebellum (Figures 1, 2 and 3), but not in corpus callosum (data not shown). Dramatic reorganization of neurovascular units was observed only in WM at or surrounding the lesion sites, but not in gray matter (Figures 1, 2 and 3). Thus fibrous astrocytes appeared to be specifically damaged in inflammatory demyelination. Both axonal demyelination and degeneration were observed in the EAE spinal cord. At the remitting stage, the lesion size and number were reduced in spinal cord, and the levels of GFAP reduced in SCWM and cerebellar GM, but not cortical GM (Figures 2, 3 and 5).

Vim is an intermediate filament and one of the markers for astrocytes. In control animals, Vim is expressed in SCWM and cerebellar Bergmann glia, but not in SCGM, cerebellar Purkinje and granule cell layers, hippocampus, and cortex (Figures 1E, 3, 4 and 5). During EAE, the level of Vim markedly increased in SCWM and hippocampus (Figures 1 and 4). Its level increased moderately in cerebellar Bergmann glia (Figure 3), and did not increase at all in other layers of cerebellum, or in cortex (Figures 3 and 5). This result shows that astrocytes that are intrinsically different respond to inflammation differently. In particular, our result that GFAP is upregulated in the hippocampus during EAE is consistent with a recent study [63]. Our finding that Vim is upregulated in hippocampal astrocytes during EAE indicates a distinct region-specific alteration in EAE. Taken together, our results add to the growing body of evidence that the hippocampus is affected in EAE, despite the lack of clear immune-cell mediated lesions characteristic of spinal cord. Recent evidence shows that in addition to astrocytes, microglia are activated in EAE [64]. The hippocampal CA1 region decreases in volume in EAE, with specific loss of GABAergic interneurons [65]. Synaptic function and immunostaining are also reduced in EAE, and can be recovered with testosterone [66] or estriol treatment [67], which are interesting results due to the gender disparity in MS incidence. Because astrocyte activation and changes in neuronal activity occur in EAE, this could correlate with the role of astrocytes in response to neuronal defects that we observed in AnkG−/− and Kv3.1−/− mice.

Increased GFAP staining around blood vessels is observed in EAE mice, which may reflect increased proliferation of perivascular astrocytes in gray matter in the brain. In a recent study using live, two-photon imaging to investigate the astrocyte response to a cortical stab-wound injury, a limited number of astrocytes (~20%) were found to proliferate, but ~70-80% of these were located immediately adjacent to blood vessels [68]. No astrocytes were found to migrate toward the lesion, but some became polarized and extended GFAP + processes toward the injury site [68]. It is unclear whether these polarized processes contributed to increased staining of perivascular astrocyte endfeet. Further study is needed to elucidate the cytokines or other secreted factors that may induce perivascular astrocyte proliferation, and how these results obtained in the local stab-wound response might correlate with the more general autoimmune insult in EAE. An interesting candidate is interferon-γ, a cytokine with major effects in MS and EAE [69] that has been shown to increase astrocyte proliferation *in vitro* and *in vivo*[70]. Moreover, a recent study indicates that in normal physiological conditions astrocytic endfeet play a major role in regulating the flow of cerebrospinal fluid and solutes into the brain parenchyma, and that AQP4 is important to this process [71]. It is possible that perivascular astrocyte proliferation may augment the astrocytic roles in fluid and solute handling and help to prevent the leakage of the BBB during EAE progression. The analysis of neurovascular units can provide more details regarding damaged nerve tissues during the progression of various neurological diseases. Although lesions were not observed in EAE in gray matter, astrocyte morphology did change, GFAP staining was increased, and astrocytes proliferated, all of which may be indicators of neuroprotective function of astrocytes.

### Neuronal activity regulates astrocytic protein expression

Purkinje neuron degeneration was observed in AnkG KO mice [36]. Highly ordered Bergmann glial processes became irregular, and GFAP staining markedly increased at the same time. The highest staining of GFAP was often associated with the loss of Purkinje cells (Figure 6B and Additional file 12: Movie S12). Kv3.1 is mainly expressed in cerebellar molecular and granule cell layers in WT mice. The highest upregulation of GFAP was found in the molecular cell layers in Kv3.1 KO mice (Figure 6C,F,H).

The patchy loss of AQP4 staining in the hippocampus of Kv3.1 KO mice may indicate the loss of astrocytes. Interestingly, this was not observed in AnkG KO mice or EAE mice at any stage (Figure 7C). This phenomenon of patchy AQP4 loss has been reported previously in the spinal cord gray matter of mice injected with AQP4 autoantibody-positive serum from NMO patients [72], but has not previously been reported in any KO mice. Kv3.1 channels are expressed in parvalbumin-positive fast spiking interneurons in the hippocampus, but it is unclear how interneuronal dysfunction in the absence of Kv3.1 could cause loss of astrocytes. A loss of astrocytes has been observed in the pilocarpine-induced epilepsy model [73], which is interesting because although very rare, we did see occasional seizures in our Kv3.1 KO mice. Indeed, synaptic transmission does appear to play a major role in regulating astrocytic protein expression.

### Differential localization of AQP4 in fibrous and protoplasmic astrocytes

AQP4 were highly colocalized with GFAP + processes in the WM, different from the pattern in the GM where it is highly concentrated in astrocytic endfeet contacting blood vessels. AQP4 expression increased in SCWM in EAE, which is consistent with a recent study in EAE and correlates with increased AQP4 levels previously shown in MS patient tissues [74,75]. This contrasts with loss of AQP4 expression in NMO, a demyelinating disease in which AQP4 autoantibodies are not only a highly specific biomarker for the disease, but are involved in disease pathology as well [76], despite some controversy for the latter [77]. In the early stage of NMO but not MS, AQP4 and GFAP are lost before the loss of MBP staining in NMO lesions, which can occur in WM or GM in mainly perivascular areas [78]. Patient MRIs also show that NMO lesions occur in both WM and GM [79]. AQP4 autoantibody-induced demyelination is thought to occur by oligodendrocyte apoptosis secondary to loss of astrocytes and astrocytic trophic support [80,81]. The BBB is more severely disrupted in NMO than in MS [82], but T cells are not required for lesion formation, as shown in mice administered IgG from NMO patients [83]. EAE susceptibility is almost eliminated in AQP4−/− mice [84,85], and secretion of proinflammatory cytokines from AQP4 −/− astrocytes was reduced *in vitro*[84]. However, AQP4 deficiency significantly increased the extent of neuron loss, demyelination, and motor dysfunction in a spinal cord contusion injury model [86]. Taken together, these results suggest that AQP4 facilitates disease progression after breakdown of the BBB, but also has a neuroprotective effect, both of which could be mediated by the central role of astrocytes in neurovascular coupling.

### Functional consequences of astrocyte activation

Astrocytes are emerging as an important player in MS, amyotrophic lateral sclerosis, Alzheimer’s disease, epilepsy, stroke, spinal cord injury, and other neurological disorders [87]. In response to a variety of CNS disorders and pathologies, astrocytes undergo tremendous changes in morphology and function, becoming activated or reactive astrocytes. These changes, including both reversible and irreversible ones, are not an all-or-none phenomenon, but are finely graded in a context-dependent manner regulated by specific signaling pathways [87]. Astrocytes exhibit excitability mainly by increasing intracellular Ca^2+^ concentration [88,89]. Astrocytes relay information to other cells through gap junctions and by releasing gliotransmitters [4,90-93]. Astrocytes promote myelination in response to electrical activity by releasing the cytokine leukemia inhibitor factor [26], or through other means, such as by coating axons with myelin-promoting extracellular matrix molecules, providing lipids for myelin synthesis, or modulating electrical activity. Various factors produced from astrocytes can also impact myelination [94], such as platelet-derived growth factor, fibroblast growth factor, ciliary neurotrophic factor, leukemia inhibitor factor, and insulin-like growth factor, as well as gliotransmitters including glutamate and ATP. Interestingly, some of these factors can further stimulate astrocytes via a positive feedback loop. Functional consequences and signaling pathways of astrocyte activation will be interesting topics for future studies in various diseases.

## Conclusions

Taken together, the analysis of neurovascular units can provide more details regarding the progression of diseases induced by either inflammatory insults or neuronal dysfunction. High-resolution confocal imaging with 3D reconstruction is a powerful tool to illustrate the changes under abnormal conditions. Better understanding their roles contributes to the development of novel strategies of neuroprotection and repair for various diseases, through activity-dependent regulation of neurovascular coupling.

## Competing interests

The authors declare that they have no competing interests.

## Authors’ contributions

PJ performed experiments, generated and analyzed imaging data, and participated in manuscript drafting and assembly. TG and VG performed experiments and provided technical support. CG conceived experiments, oversaw data collection and analysis, and performed manuscript drafting and assembly. All authors read and approved the final manuscript.

## Supplementary Material

Additional file 1: Movie S1 An image stack from a longitudinal section of SCWM of a control Thy1-YFP mouse. YFP labels a subset of axons. GFAP staining is in blue and AQP4 staining in red. This is the supplemental movie for Figure 1G. Click here for file

Additional file 2: Movie S2 An image stack from a longitudinal section of SCWM of an EAE Thy1-YFP mouse. YFP labels a subset of axons. GFAP staining is in blue and AQP4 staining in red. This is the supplemental movie for Figure 1H. Click here for file

Additional file 3: Movie S3 An image stack from a coronal section of the cerebellar molecular layer in a control mouse. GFAP staining is in green, AQP4 staining in red and Vim staining in blue. This is the supplemental movie for Figure 3A. Click here for file

Additional file 4: Movie S4 An image stack from a coronal section of the cerebellar molecular layer at the peak stage of rrEAE. GFAP staining is in green, AQP4 staining in red and Vim staining in blue. This is the supplemental movie for Figure 3B. Click here for file

Additional file 5: Movie S5 An image stack from a coronal section of the cerebellar molecular layer at the remitting stage of rrEAE. GFAP staining is in green, AQP4 staining in red and Vim staining in blue. This is the supplemental movie for Figure 3C. Click here for file

Additional file 6: Movie S6 An image stack from a coronal section of the cerebellar molecular layer at the relapsing stage of rrEAE. GFAP staining is in green, AQP4 staining in red and Vim staining in blue. This is the supplemental movie for Figure 3D. Click here for file

Additional file 7: Movie S7 An image stack from a coronal section of the hippocampus in a control Thy1-YFP transgenic mouse. YFP fluorescence is in green, AQP4 staining in red and GFAP staining in blue. This is the supplemental movie for Figure 4D. Click here for file

Additional file 8: Movie S8 An image stack from a coronal section of the hippocampus in a Thy1-YFP transgenic mouse at the peak stage of chEAE. YFP fluorescence is in green, AQP4 staining in red and GFAP staining in blue. This is the supplemental movie for Figure 4E. Click here for file

Additional file 9: Movie S9 An image stack from a coronal section of the cerebral cortex in a control Thy1-YFP transgenic mouse. YFP fluorescence is in green, AQP4 staining in red and GFAP staining in blue. This is the supplemental movie for Figure 5D. Click here for file

Additional file 10: Movie S10 An image stack from a coronal section of the cerebral cortex in a Thy1-YFP transgenic mouse at the peak stage of chEAE. YFP fluorescence is in green, AQP4 staining in red and GFAP staining in blue. This is the supplemental movie for Figure 5E. Click here for file

Additional file 11: Movie S11 An image stack from a coronal section of the molecular layer of cerebellar cortex in a WT mouse. PKCγ staining is in green and GFAP staining in red. This is the supplemental movie for Figure 6A. Click here for file

Additional file 12: Movie S12 An image stack from a coronal section of the molecular layer of cerebellar cortex in an AnkG KO mouse. PKCγ staining is in green and GFAP staining in red. This is the supplemental movie for Figure 6B. Click here for file

Additional file 13: Movie S13 An image stack from a coronal section of the molecular layer of cerebellar cortex in a Kv3.1 KO mouse. PKCγ staining is in green and GFAP staining in red. This is the supplemental movie for Figure 6C. Click here for file

Additional file 14: Movie S14 An image stack from a coronal section of the hippocampus in a WT mouse. GFAP staining is in green and AQP4 staining in red. This is the supplemental movie for Figure 7A. Click here for file

Additional file 15: Movie S15 An image stack from a coronal section of the hippocampus in an AnkG KO mouse. GFAP staining is in green and AQP4 staining in red. This is the supplemental movie for Figure 7B. Click here for file

Additional file 16: Movie S16 An image stack from a coronal section of the hippocampus in a Kv3.1 KO mouse. GFAP staining is in green and AQP4 staining in red. This is the supplemental movie for Figure 7C. Click here for file

Additional file 17: Movie S17 An image stack from a coronal section of the cerebral cortex in a WT mouse. GFAP staining is in green and AQP4 staining in red. This is the supplemental movie for Figure 7D. Click here for file

Additional file 18: Movie S18 An image stack from a coronal section of the cerebral cortex in an AnkG KO mouse. GFAP staining is in green and AQP4 staining in red. This is the supplemental movie for Figure 7E. Click here for file

Additional file 19: Movie S19 An image stack from a coronal section of the cerebral cortex in a Kv3.1 KO mouse. GFAP staining is in green and AQP4 staining in red. This is the supplemental movie for Figure 7F. Click here for file
